# Commentary: SARS-CoV-2 and Asbestos Exposure: Can Our Experience With Mesothelioma Patients Help Us Understand the Psychological Consequences of COVID-19 and Develop Interventions?

**DOI:** 10.3389/fpsyg.2021.720160

**Published:** 2021-09-09

**Authors:** Daniela Di Basilio, Jun Shigemura, Fanny Guglielmucci

**Affiliations:** ^1^Department of Psychology, Manchester Metropolitan University, Manchester, United Kingdom; ^2^Faculty of Health Sciences, Mejiro University, Saitama, Japan; ^3^Department of Psychology, University of Turin, Turin, Italy; ^4^Laboratory of Research and Interventions in Psychoanalysis (psiA), Department of Clinical Psychology, Institute of Psychology, University of São Paulo, São Paulo, Brazil

**Keywords:** COVID-19, asbestos, ecological trauma, trauma interventions, risk prevention, post-disaster trauma, public health

Recently, Granieri et al. (2020) highlighted multiple similarities between COVID-19 and Malignant Mesothelioma (MM). Here, we argue that despite the relevant similarities between these two conditions, their differences—including aetiology, infection pattern, chronological course, physical symptoms, and prognosis—outweigh their similarities. Moreover, we suggest the need to move away from a mere symptom consideration, adopting an ecological perspective focused on the different levels on which trauma occurs and the intra- and inter-personal dynamics involved in these diseases.

Firstly, as the Authors suggested, there is an important difference between the causative processes in MM and COVID-19, which reflects on their mental representations and dynamics. As asbestos exposure is usually connected to industrial activity, MM can be characterised as an occupational disease (Noonan, 2017), for which responsibility and intentionality can be traced in companies, purposely exposing their workers to harmful pollutants and prioritising their profit instead of people's safety (Guglielmucci et al., 2014). MM patients often feel betrayed by their employers, with consequent anger and claims for compensation (Sherborne et al., 2020). These behaviours could be seen as an attempt to minimise feelings of guilt over one own's responsibility for negative health outcomes, to find an external scapegoat to blame and to increase the perceived control over a situation entailing feelings of danger and helplessness (Rothschild et al., 2012; Guglielmucci, 2016).

Conversely, COVID-19 is a pandemic of probable zoonotic origin (Ivers and Walton, 2020)—despite the controversies about its origins (Mallapaty et al., 2021)—and there is, therefore, no identifiable culprit. COVID-19 has represented a global mental health crisis [World Health Organization (WHO), 2020a for a large-scale meta-analysis of the evidence of COVID-19 impact on public mental health services, see Liu et al., 2021] leading to widespread negative consequences on the mental health and well-being of individuals, communities, and societies (Dong and Bouey, 2020; Kazlauskas and Quero, 2020; Shigemura et al., 2020) and financial and economic disruption (Pak et al., 2020; World Health Organization (WHO), 2020b). In other words, when considering COVID-19, we need to take into account a *systemic* dimension of trauma and the presence of a violation of social trust. The lack of clear information about the disease's transmission and the available treatments have contributed to promoting uncertainty, fear of dying and fear of others, perceived as a possible vehicle of infection (Presti et al., 2020; Schimmenti et al., 2020). Despite—as the Authors argue—similar dynamics related to the fear of an “aerial contagion” brought by an “invisible killer” and the fear to infect/to be infected by others have also been found to be related to MM (Guglielmucci et al., 2015, 2018), these dynamics are mainly found in people residing in contaminated sites, in which asbestos exposure assumes a wider resonance as it potentially applies to the whole community.

Additionally, the traumatic breadth of the COVID-19 situation was enhanced by the social restrictions imposed by authorities, which did not occur in MM and have often caused people to experience loss of social interactions and income, increasing the prevalence rates of psychological problems all over the world (see for example Ahrens et al., 2021; Evans et al., 2021).

In emergency and crisis situations, people often turn to authorities to receive guidance and support and the quality of their responses can influence how the public copes with hardship (Smith, 2006; Glik, 2007). Since the start of the pandemic, individuals have relied on governments, services, and institutions which have often failed to recognise these vulnerabilities in their citizens and to respond appropriately to their needs (Altman, 2020; Karlsson, 2020; Olivia et al., 2020; Ham, 2021), often worsening socio-economic inequalities (Dorn et al., 2020) and betraying people's needs for security, protection, and care (Klest et al., 2020). This responsiveness failure constitutes a type of traumatic experience known as “institutional betrayal,” which refers to wrongdoings perpetrated by institutions upon individuals relying on them and include failure to prevent and respond appropriately to individuals' needs (Smith and Freyd, 2013, 2014). All these dynamics put to test—consciously or unconsciously—the capability of the mind to deal with feelings of powerless and helplessness, exposing people to long-lasting detrimental psychological effects that may even influence future generations (Leuzinger-Bohleber and Montigny, 2021).

Therefore, whilst the traumatic sequelae of MM are mainly situated at an *individual* and *community* level, the ones related to COVID-19 can be best understood as occurring at a *systemic level*, as they led to a cumulative, collective/societal trauma affecting the relationships among individuals and between individuals and their “macrosystem” (Scalabrini et al., 2020).

In Bronfenbrenner's 1979 ecological model, the “macrosystem” encompasses -among others- cultural influences, media messages, social policies, economic systems, government agencies, educational, and healthcare resources. This considered, to better understand the differences between the type of trauma (and its sequelae) in MM and COVID-19, we propose a model named “Ecological Response to Complex Trauma (ERCT) model (Figure 1).”

**Figure 1:**
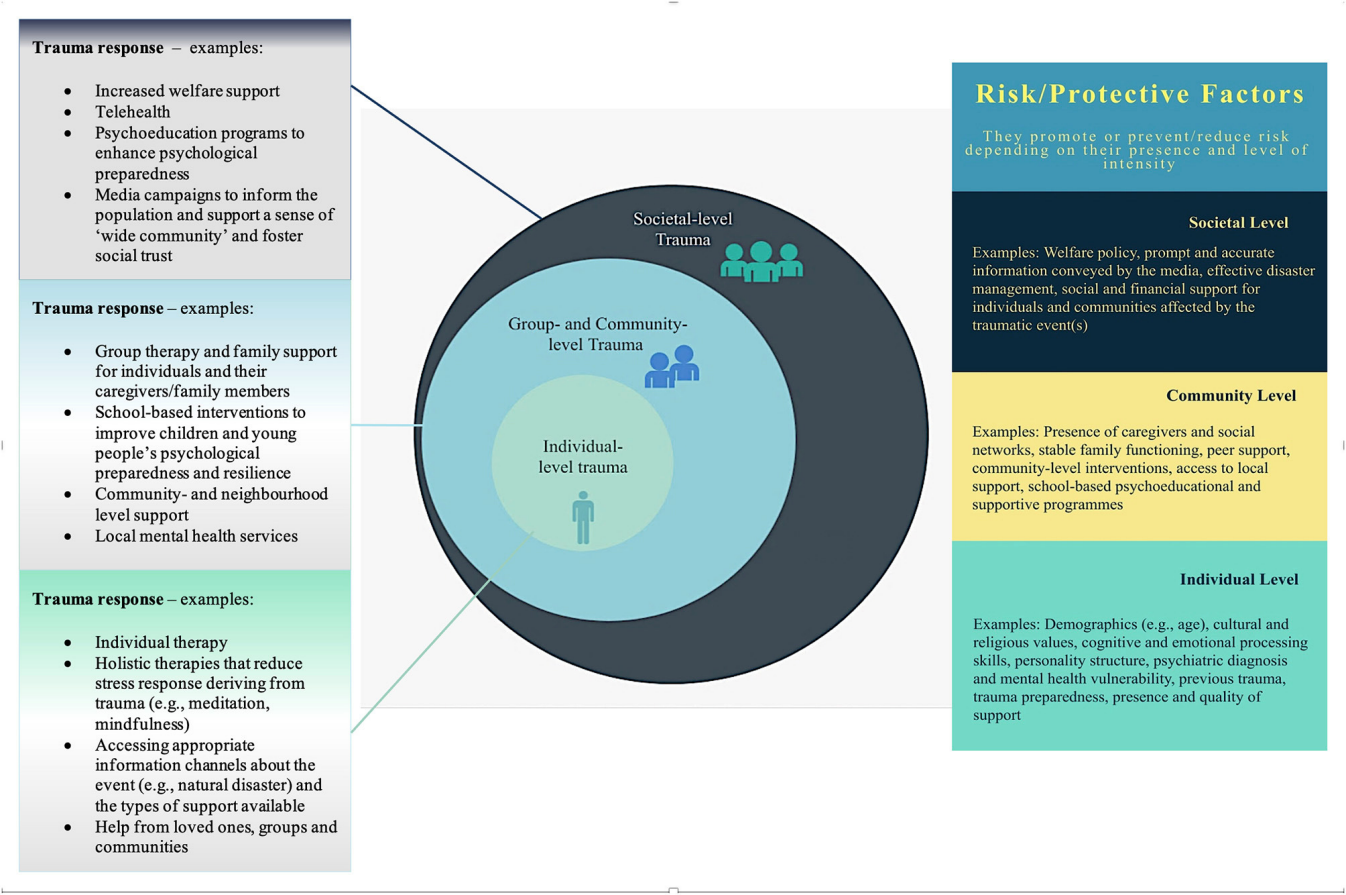
Ecological Response to Complex Trauma (ERCT) model.

The ERCT model entails three concentric levels (individual, community, and societal) that trauma can occur on. For each level, there are specific mental representations of the traumatic situation and risk/protective factors, which are intertwined and create cross-pathways among levels. We also suggest some of the measures that should be present on each level to mitigate the impact of trauma. For example, some of the individual-level protective factors include emotional and cognitive preparedness to face a natural or man-made disaster (Gabriel et al., 2007; Roudini et al., 2017), absence of pre-existing trauma and psychiatric history (Alvarez and Hunt, 2005; Esterwood and Saeed, 2020), good emotional regulation skills (Restubog et al., 2020; Wang et al., 2021), and sense of control during and after the disaster (Reich, 2006). At collective and societal levels, examples of protective factors include social support (Ehring et al., 2011; Huang et al., 2013; Pietrzak et al., 2014), community-level preparedness training (Morrissey and Reser, 2003), receiving post-disaster professional support (Tak et al., 2007; Brooks et al., 2016) and using social media as a source of information and psychological “first aid” (Finch et al., 2016; Yang et al., 2019). Social media were also used to cope with social isolation and feelings of loneliness in COVID-19 lockdown phases (Boursier et al., 2020), providing an “online community” that improved people's collective resilience (Marzouki et al., 2021). Previous literature on collective/social traumatic events (e.g., Eriksson, 2015; Neubaum et al., 2014) showed that in disaster contexts, social media can be useful for social regulation and information sharing. Nonetheless, during COVID-19 social media also contributed to spreading panic (Ahmad and Murad, 2020), and in some cases, the fear of “missing out” and becoming “socially invisible” resulted in excessive use of online social interactions (Gioia et al., 2021), showing a dual-sided connotation of social media engagement as both able to foster relational closeness and alleviate social panic (Wiederhold, 2020; Cauberghe et al., 2021; Musetti et al., 2021) and as a maladaptive behaviour leading to increased levels of anxiety and negative affect contagion (Boursier et al., 2020; Shao et al., 2021).

Clinical implications of our model also are relevant for targeting psychological interventions. In our model, MM could be conceptualised as a two-level traumatic event affecting MM patients and families—in line with Granieri et al. (2020), group therapy could be beneficial to address the effects of MM-related trauma. Conversely, as COVID-19 is a social catastrophe, it would be best addressed with a combination of interventions encompassing the three trauma levels. In other words, when in presence of traumatic events affecting the individual, community and societal levels, the type of *trauma response* should also be complex and multi-level. This implies that social agents (e.g., governments, policymakers, social media) should acknowledge their responsibility in supporting trauma containment and trauma healing and show responsiveness particularly in two main domains: (1) Healthcare and (2) Informative communication to foster education and trauma preparedness. More specifically, a wider range of need-based, person-centred interventions for different target populations (e.g., COVID-19 survivors, their family members, healthcare professionals, and the general public) should be provided, with specific consideration for the socio-economic determinants of health that may increase risk or provide protection against mortality, morbidity, and trauma-related outcomes (Abrams and Szefler, 2020; Burström and Tao, 2020). These interventions should reach a large number of individuals in a relatively short time, with particular attention to the most vulnerable segments of society (e.g., immigrants, ethnic minorities, people with unstable and low-income jobs), to mitigate pre-existing socioeconomic disparities and the pandemic-related burden (Bambra et al., 2020; Dorn et al., 2020).

Digitally delivered care and telehealth (e.g., video calls or app-delivered support) have the potential to achieve these aims, fostering a considerable sense of control over one's health, and facilitating access to services (McGeary et al., 2012; Keshvardoost et al., 2020). Telehealth and digital interventions have proven to be beneficial to those experiencing mental health issues during human-caused and natural disasters (Ruzek et al., 2016) and recent evidence (Centers for Disease Control Prevention, 2020; Monaghesh and Hajizadeh, 2020) indicated that their use increased exponentially during COVID-19. Moreover, in societal-level and global crises, governments and institutions should pay particular attention to the communicative strategies adopted, to avoid the confusing messages and panic-eliciting communication that occurred during the COVID-19 pandemic (Garrett, 2020; Sauer et al., 2021).

On the contrary, trauma-informed communication should entail the use of media (including social media) to contain negative emotions, “buffer” the traumatic response and help to maintain a sense of social trust, which is likely to be deeply compromised in situations of systemic-level trauma (Bachem et al., 2020).

In conclusion, the ERCT model proposes that complex trauma such as the one characterising COVID-19 needs to be addressed with multi-level trauma-informed care. Governments and institutions should be more aware of the dynamics at play and of their role in them. A structural change in our society is needed, “transitioning from a primary disease-centred system to a balanced preventive and healthcare system” (Fontana et al., 2021, 4). Discussions should be held on how to engage current and future generations to allow for this “switch” to happen. In our opinion, health literacy rooted in the complex array of relations among physical, socio-economic, affective, and environmental dimensions that can worsen or mitigate trauma effects should be embedded in education pathways across the life span (and particularly in early stages) to shape health and well-being across people's lives and promote appropriate individual, community, and systemic responses to trauma.

## Author Contributions

DD wrote the manuscript, led the literature research, conceived the clinical-conceptual model in collaboration with FG, and designed the figures with input from all authors. FG co-produced the clinical-conceptual model and critically revised the manuscript providing important theoretical and clinical contributions. JS contributed to the writing of the manuscript offering feedback and valuable advice throughout the writing process. All authors contributed to the article and approved the submitted version.

## Conflict of Interest

The authors declare that the research was conducted in the absence of any commercial or financial relationships that could be construed as a potential conflict of interest.

## Publisher's Note

All claims expressed in this article are solely those of the authors and do not necessarily represent those of their affiliated organizations, or those of the publisher, the editors and the reviewers. Any product that may be evaluated in this article, or claim that may be made by its manufacturer, is not guaranteed or endorsed by the publisher.
